# Triangular Sierpinski Microwave Band-Stop Resonators for K-Band Filtering

**DOI:** 10.3390/s23198125

**Published:** 2023-09-27

**Authors:** Romolo Marcelli, Giovanni Maria Sardi, Emanuela Proietti, Giovanni Capoccia, Jacopo Iannacci, Girolamo Tagliapietra, Flavio Giacomozzi

**Affiliations:** 1 CNR-IMM, 00133 Roma, Italy; giovannimaria.sardi@cnr.it (G.M.S.); emanuela.proietti@cnr.it (E.P.); giovanni.capoccia@cnr.it (G.C.); 2 Fondazione Bruno Kessler, Povo, 38123 Trento, Italy; iannacci@fbk.eu (J.I.); gtagliapietra@fbk.eu (G.T.); giaco@fbk.eu (F.G.)

**Keywords:** Sierpinski triangle, microwave resonators, frequency tunability, metamaterials

## Abstract

Triangular resonators re-shaped with Sierpinski geometry were designed, manufactured, and tested for potential applications in the K-Band. Prototypes of band-stop filters working around 20 GHz and 26 GHz, interesting for RADAR and satellite communications, were studied in a coplanar waveguide (CPW) configuration. Single and coupled structures were analyzed to give evidence for: (i) the tuning of the resonance frequency by increasing the internal complexity of the triangle and (ii) resonance enhancement when coupled structures are considered. The exploited devices were part of the more extended family of metamaterial-inspired structures, and they were studied for their heuristic approach to the prediction of the spectrum using experimental results supported by electromagnetic simulations. As a result, a Sierpinski resonator, not only fed into but also fully embedded into a CPW environment, had a frequency response that was not easily determined by classical theoretical approaches.

## 1. Introduction

Sierpinski triangles are fractal complex configurations obtained by defining empty sub-triangles inside a full triangle. Since the seminal work in [1], several contributions have been published regarding 2D and 3D configurations and their applicability in different fields. It must be stressed that the merit of Sierpinski geometry is to give a mathematical formulation of the problem, even if the same geometries are easily found for decorative purposes in ancient monuments, such as the floors of many churches or even columns in cloisters, and studied from cultural heritage and mathematical perspectives [2,3].

In the case of microwave planar resonators, to obtain a Sierpinski structure means to include empty triangles inside a metal triangular full patch. Some examples of the multi-resonant response of these configurations and their properties for high-frequency signal processing have been published, especially regarding antenna applications [4,5,6,7]. Still, resonators inspired by the same geometry have been considered [7,8]. A preliminary study on some Sierpinski resonators and their metamaterial nature was also published by us [9].

Our contribution aims to study a specific fractal geometry and its high-frequency response using a coplanar waveguide configuration for a planar resonator. Many research articles on the Sierpinski geometry have been focused on free propagation structures and their utilization for multiband purposes, but few contributions are available for planar signal processing. Among others, we also selected recent studies [10,11]. The results from [10] mainly focused on traditional coupled line filters mixed with Sierpinski triangles to fine-tune the filter response. In the paper, the results consisted of the multi-band response and some improvement in the component footprint. Additional properties were considered for wideband applications, such as the lowering of the operative frequencies and better electrical matching, linking the resonant narrowband response of the triangles to the wideband performance of the coupled line filter. In [11], a completely different geometry was considered, still inspired by Sierpinski geometry, and focused on a resonant narrowband response but in a gasket configuration, potentially to be manufactured on a suspended membrane.

Our paper presents a complete study of triangular equilateral resonators, with a systematic experimental approach to the single and coupled structures inspired by Sierpinski geometry. The obtained results should be considered the basis for a heuristic approach to interpret the response of fractal microwave Sierpinski resonators in coplanar configuration. We give evidence for what can be easily determined and the difficulties in efficiently exciting the Sierpinski configurations or predicting their resonance frequencies compared to microstrip-fed structures.

In the following sections, we analyze some Sierpinski triangular structures designed and manufactured on oxidized high-resistivity silicon wafers and measured on-wafer. We compare the measurement with the theoretical expectations obtained using electromagnetic 2.5D commercial software. Simulations were performed using the AXIEM environment of the AWR-CADENCE Microwave Office software release 22.1, and the tests were carried out with the on-wafer measurement system available at CNR-IMM. The devices were manufactured using the technological facilities of FBK. The operating frequencies *F* originally selected for the studied resonators were 20 GHz and 26 GHz, respectively. They lie in the K-band (18–27 GHz), currently, one of the frequency ranges most used for satellite telecommunications. The Ku (K-under, 12–18 GHz) and Ka (K-above, 27–40 GHz) bands were also used for the same purposes. Typical requirements for transmit/receive (TX/RX) signals, i.e., for uplink (towards the satellite) and downlink (back to the earth) operations, are around 30 GHz and 20 GHz, respectively. An uplink frequency higher than the downlink one is justified by a lower noise contribution and lower losses in the atmosphere for the propagation of waves with lower frequency. Using two different frequencies helps prevent oscillations generated by the satellite amplifier when simultaneously processing both signals.

In this framework, the novelty of this paper lies in the systematic investigation of fractal Sierpinski resonators excited by a coplanar waveguide and coupled structures made by the side coupling of individual triangles instead of using the microstrip-fed resonators, already studied and currently available in the literature. Moreover, we focus our attention on the guided propagation response, while many papers have proposed structures useful for free propagation (antennas). Considerations are drawn regarding the spectrum interpretation and the necessity of refinement in determining the frequency of resonance, which cannot only be predicted by the effective side length of the triangle, such as in the case of microstrip excitation [12]. A simple perturbation approach is not enough to predict the excited modes when considering the increasing complexity of the Sierpinski triangles.

## 2. Spectral Considerations of Sierpinski Resonators

A complete analytical approach to determining the Sierpinski geometry and designing triangles is available in several books, papers, or mathematical packages [13,14]. Nevertheless, it is a complex task to determine the frequency of resonance when the same triangles are utilized as electromagnetic resonating structures. Only the main resonance frequency *F_resonance_* for equilateral full triangles (or simple geometries such as isosceles and right-angled triangles) was exactly calculated, considering the TM (Transverse Magnetic) mode configuration for the equivalent cavity model. The following formula gives the resonance frequency *F_resonance_* for the main mode of the equilateral triangle [12,15,16,17], as it was also used in [9]:

(1) $$F_{resonance}=\frac{2c}{3a_{effective}\sqrt{\varepsilon_{effective}}}$$

where *c* is the light speed, and the effective values of the triangle edge *a* (*a_effective_*) and the relative dielectric constant *ε* (*ε_effective_*) might be introduced, as in [12], to account for the needed phenomenological corrections typically adopted for planar high-frequency geometries. It must be remembered that, according to the results in [12], only the effective value of the side length (*a_effective_*) of the triangle is necessary to have an agreement between the experimental value of the frequency of resonance and the expected one for the main mode given by Equation (1).

It has already been discussed in [9] why triangular geometry is advantageous in terms of higher frequencies, array configuration, and footprint compared to circles and squares. For completeness, we recall that the main advantages of using triangles compared to squares are the smaller footprint and a higher resonance frequency. Using basic formulas to determine *F_resonance_*, it is expected to be in a ratio of 4/3 using a triangle with the same edge length as a square. At the same time, the area reduction is in the order of 0.4, which is a clear advantage when integrating the resonators into a planar array. Circles are easier to design but difficult to integrate using a side-by-side coupling. The disadvantage with triangles is the necessity for an accurate design accounting for the discontinuities on the corners.

**Figure 1:**
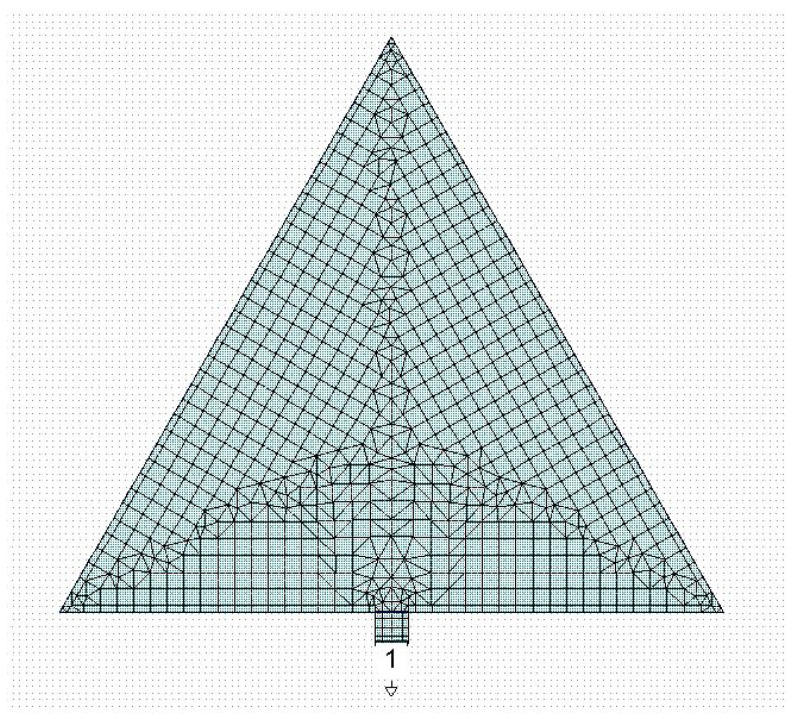
Full equilateral triangle (C0) excited by a microstrip placed in the center of the side and its meshing used for the simulation. The number “1” is the reference port of the simulation, while the external small triangle indicates that the port is grounded.

An efficient excitation of the resonator’s modes implies the proper coupling between the resonator and the signal launcher. Not all the resonance modes can be efficiently excited because the maximum of some modes could be far from the excitation point. How excited should the resonance modes of a triangle be? Many authors have studied triangular antennas with a via hole close to the vertex of the patch triangle, fed by a microstrip lying on the backside of the substrate. The via hole is at a distance *d* to the vertex. We underline that there is no published general criterion to precisely determine where the via hole should be positioned depending on the resonance mode to be excited. For this reason, the frequency of resonance of the first few modes has been analytically calculated and experimentally measured in the literature, but the spectrum is rarely shown, probably because the modes are not evidenced due to poor electrical matching. A simple way to feed the triangular resonator by a microstrip on the same substrate where the triangle has been manufactured, instead of using a via hole, is given in Figure 1, where a 50-ohm short line is connected to the edge of the resonator. The conventional labels used in this paper to define different Sierpinski resonators are C0 for the full patch. At the same time, C1, C2, and C3 are concerned with sub-divisions up to the third level of internal complexity.

**Figure 2:**
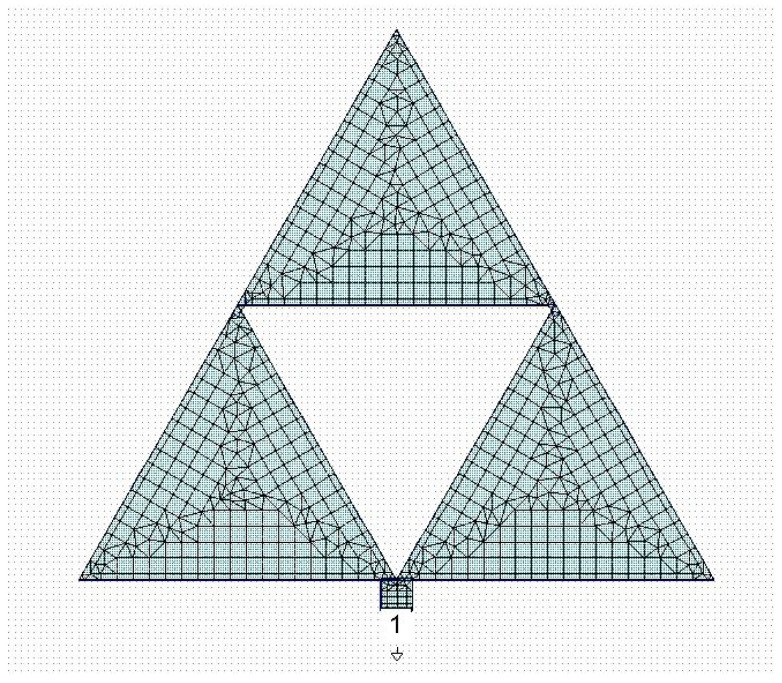
First-order Sierpinski triangle excited by a microstrip and the meshing used for the simulation. The number “1” and the downward external small triangle are the port number for the simulation and its ground reference, respectively.

The same arrangement was used for the Sierpinski triangle with complexity C1; the results are shown in Figure 2. In both cases, we used, for the simulation performed using the CADENCE Microwave Office Software release 22.1, the same geometry and materials considered in the 1987 paper regarding the spectrum of an equilateral triangular and the influence of effective values for the dielectric constant and the edge side [12]. In the paper, the conclusion was that the theory matches the experiment when an effective side length is considered without changes in the formulas for the dielectric constant. Then, the substrate was the Rogers product RO5870 with ε=2.32, and a thickness of t=1590 μm.

The 50-ohm microstrip was designed with a width of W=4700 μm. The substrate is grounded, and the input/output port is referred to that ground.

**Figure 3:**
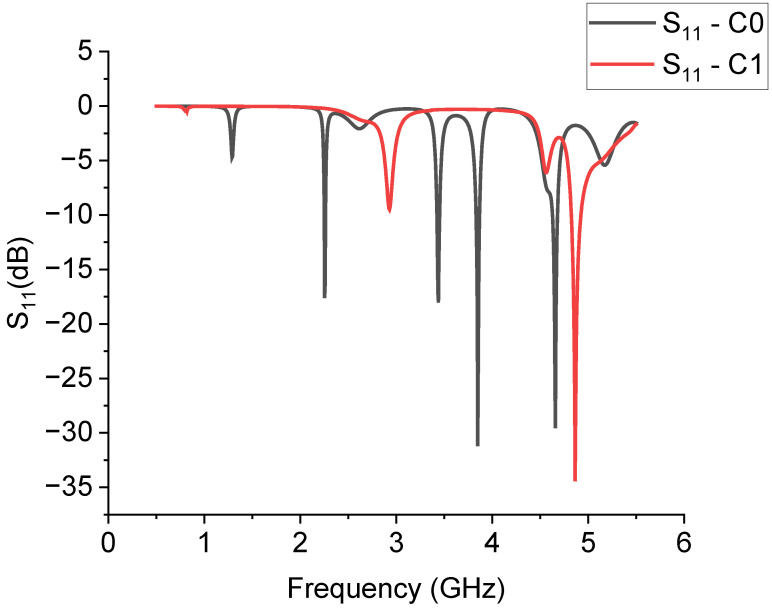
Spectral response of the triangles C0 and C1 excited by a short microstrip connected to the edge of the triangles. The dark curve is for C0 and the red one is for C1.

The spectral response obtained for C0 and C1 by the simulation is shown in Figure 3, where the simulated scattering (S) parameter in the reflection, i.e., the matrix element S_11_, is plotted on a dB scale.

From the spectral analysis, we can argue two main facts: (a) the excitation of the modes is much more efficient for the full triangle C0 than the C1 configuration, and (b) the excited modes for the C0 resonator agree very well with the measured ones in [12], as shown in Table 1.

For an error not exceeding 2%, calculating the quantity $err=|f_{sim}-f_{exp}|/f_{exp}$ is evaluated, where $f_{sim}$ is the simulation result and $f_{exp}$ is the experimentally measured frequency of resonance from [12]. The third mode gives the worst case, but it is the one excited with the lowest efficiency.

**Figure 4:**
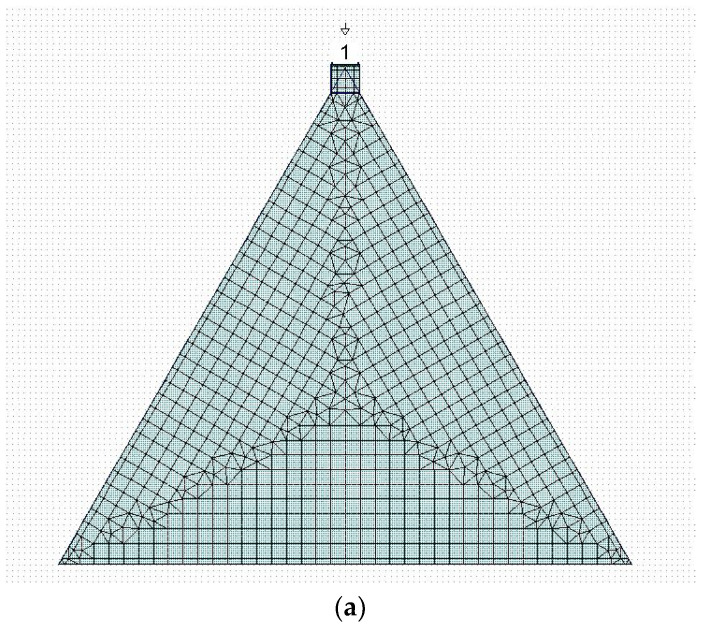

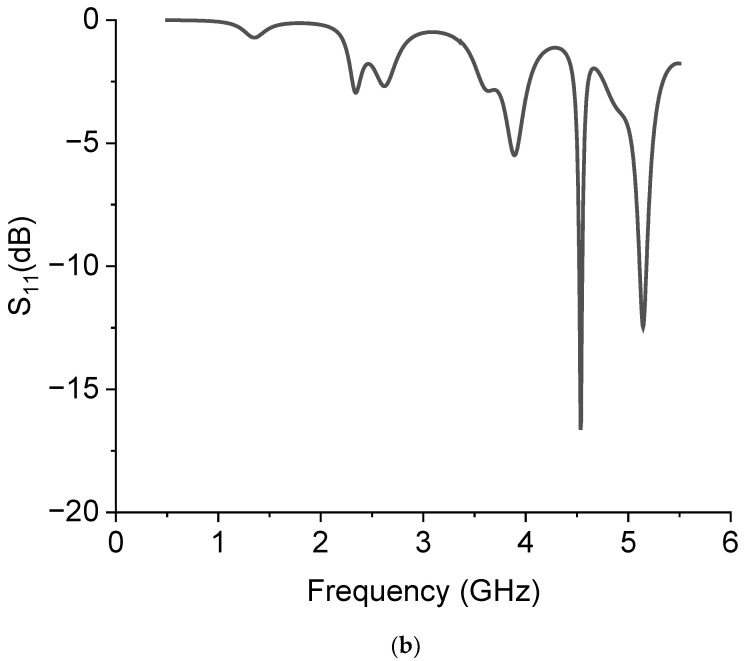
Excitation by microstrip of the C0 configuration using a corner of the triangle (**a**), and its spectral response using the reflection parameter S_11_ (**b**). The number “1” in (**a**) is the port number for the simulation and the triangle is the ground reference, respectively. The meshing used for the simulation is also shown in (**a**).

The interpretation of the resonance modes is more difficult when we analyze the C1 configuration. In the same frequency range used for simulating C0, only two modes are clearly visible for C1, and a third one is depressed by the proximity with a more intense mode, around 4.56 GHz. It should be stressed that this comparison was performed by changing the excitation system of the triangle with respect to [12], but a full agreement was obtained for C0. To complete the analysis, the following Figure 4 shows how the position of the excitation point of the C0 structure, placed on the corner of the triangle, affects the resonance. The spectrum is almost the same but with a less efficient excitation of the resonance modes and broader peaks because of the utilization of a discontinuity point to feed the resonator.

There are a wide range of instances in the literature regarding Sierpinski fractals for antennas, but most of the contributions are, as in our case, the result of systematic simulations and experimental efforts to reconstruct the spectrum. A good overview of the guidelines that should be followed for designing a fractal antenna based on Sierpinski geometry can be found in [18,19], also inspired by the seminal review in [20].

The papers suggested a fitting procedure using electromagnetic simulations and experimental measurements. The authors of [18,19] were inspired by the necessity to have formulas for practical purposes, and the simulation and experimental efforts produced clear guidelines for antennas in a microstrip-fed structure. As a result, valuable formulas have been published for this specific configuration to provide a reliable numerical approach to the design of triangular antennas. Again, this is a different finding than our efforts because triangular antennas were studied in those papers and fed using a microstrip.

**Figure 5:**
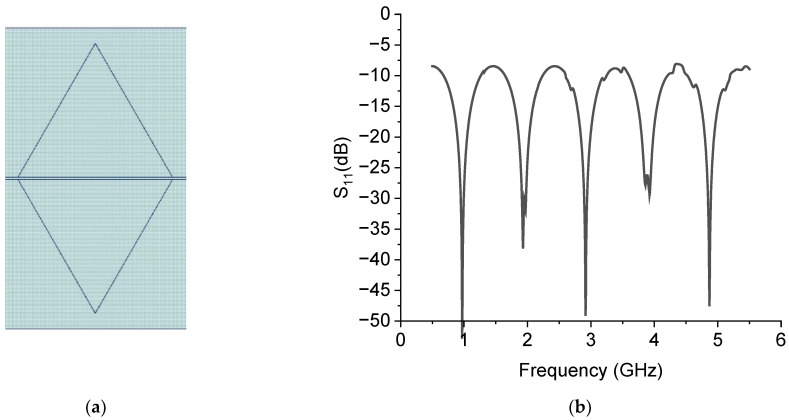
CPW excited C0 resonator (**a**) and its microwave response in reflection (S_11_ parameter) (**b**).

Using a CPW to obtain a triangular resonator implies an excitation obtained by side-coupling the triangle with the central conductor and a ground plane surrounding the geometrical figure. A CPW allows for an in-plane electric field, and it is convenient for filters to be manufactured on a stack of non-interacting planes. A preliminary evaluation of the C0 structure using the same RO5870 substrate was attempted with a CPW designed to have a 50-ohm transmission line exciting the resonator again. For this purpose, the CPW structure was composed of a central conductor 1200 μm wide with a gap 50 μm wide. The gap size was selected to be equal to the structures that were successively manufactured on silicon, placing the distance between the triangle side and the lateral ground as the CPW gap. At the same time, the width of the central conductor was scaled to account for a 50-ohm line. The resonator was symmetrically placed in the CPW to improve the efficiency of the electrical coupling, as discussed later. In Figure 5, the designed structure and its spectral response to the reflection scattering parameter S_11_ are plotted.

Analyzing Figure 5, it turns out that the excited spectrum is similar to that obtained with microstrip feeding, except for two major differences: (i) the frequencies of resonance are shifted up or down, and (ii) a good value for the depth of the peak is obtained, but an impedance transformation is needed at the I/O ports to improve the zero-level response of the device. The experienced changes in the frequencies of resonance in comparison with the microstrip configuration, using data from Table 1 and the following Table 2, are difficult to categorize as a simple up-shift or down-shift, because the excitation configuration can have an important role in the manifestation of specific resonance modes. A more detailed theoretical electromagnetic analysis, eventually followed by purposely designed prototypes, is reserved for further contributions to the same topic, studying from theoretical and experimental perspectives the critical role of the feeding in the effective excitation of the CPW Sierpinski resonators. For now, we designed and manufactured devices only with the choice of side coupling according to the typical CPW layout. In fact, except for the first two modes, downshifted by approximately 320 MHz compared to those excited by a microstrip, the following three modes are not comparable in an easy way. This finding suggests that an equivalent circuit and an electromagnetic formulation of the triangular patches must be re-calculated to have a reliable theory to predict the frequencies of resonance when a CPW configuration feeds the triangular resonator.

## 3. Design of the Single 20 GHz Resonators

**Figure 6:**
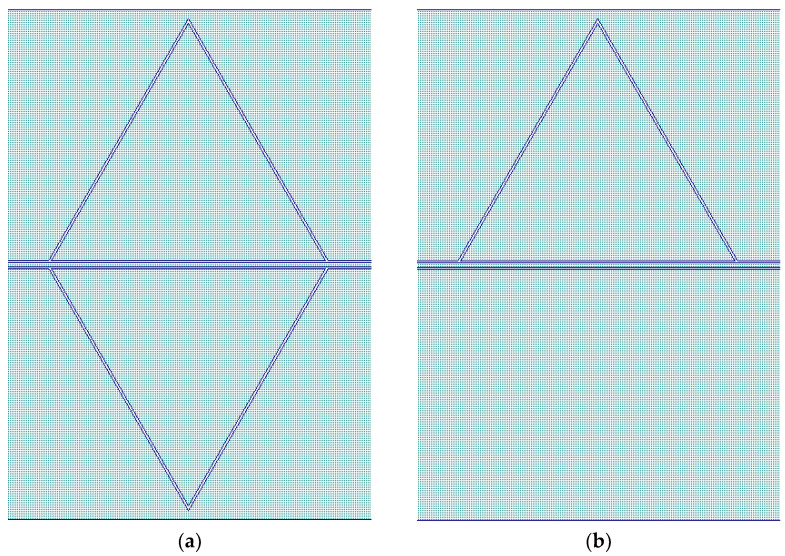

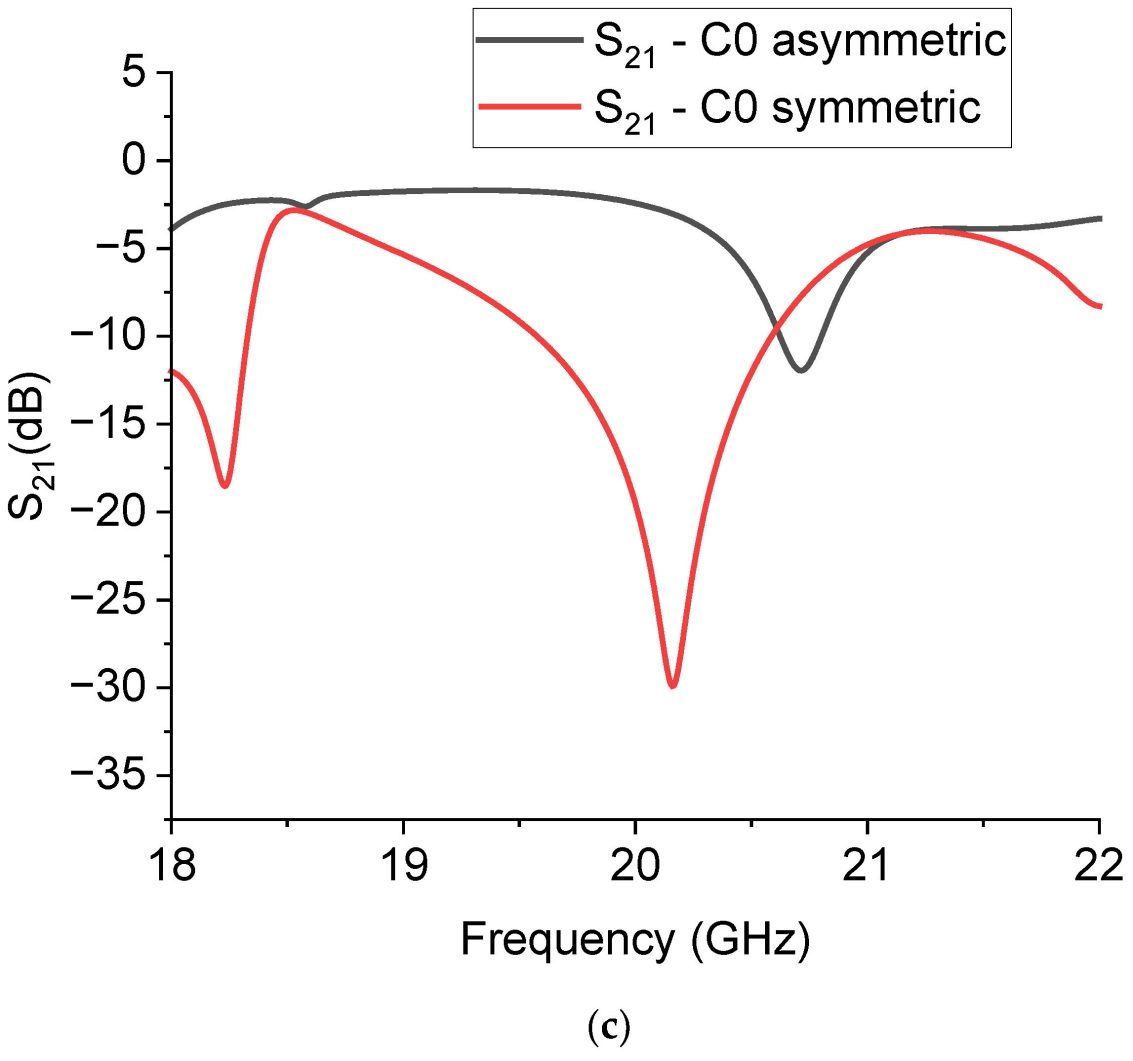
Symmetric (**a**) and asymmetric (**b**) configurations of a triangular patch resonator in the CPW configuration, and comparison (**c**) between the expected performances of the transmission S_21_ parameter of both structures in dB scale.

Single Sierpinski microwave resonators were designed in a coplanar waveguide (CPW) configuration for band-stop filtering to take advantage of the confinement of the electromagnetic field mainly in the plane of the wafer, which allows the studied structures to be also suitable for realization on multiple planes without mutual interference. The simulations were performed with the CADENCE-AWR Microwave Office Software release 22.1. The CPW symmetry, with one central conductor and two lateral ground planes, imposes a symmetrical arrangement of the resonating structure, which is the main difference for the same resonators excited by microstrip transducers. The above consideration is demonstrated in Figure 6, where single and doubled patch triangles were simulated for the 20 GHz operation. The best impedance matching (with respect to the 50 Ω I/O lines) is displayed by the red curve, related to the transmission parameter S_21_ of the symmetric structure; i.e., when the peak of the band-stop filter is deeper.

The CPW was designed as a wideband transmission line with the central conductor 80 μm wide and the slot 50 μm wide. The choice for the side length of the triangle was initially made in agreement with Equation (1), accounting for the necessary recalculation of the geometrical side length *a* from the effective one, in order to obtain the envisaged frequencies. On the other hand, this equation was unsuccessful in having a good prediction of *F_resonance_* for the case of the CPW-fed resonators, even considering that the CPW has been manufactured onto an oxidized high resistivity Silicon wafer, and accounting for a dielectric constant value of approximately ε=5.5 for a wide frequency range, as obtained in [21]. For this reason, we tried values of *a* busing electromagnetic simulations to obtain operating frequencies close to the desired ones, choosing $a\left(20\;GHz\right)=6\;mm$ and $a\left(26\;GHz\right)=4\;mm$. As a result, the C0 configuration was simulated almost in agreement with the desired value of *F_resonance_*, while we saw that higher frequencies needed further elaboration to improve both the prediction of the resonance and the selectivity of the resonating structure. An additional comment must be made regarding the symmetry of the boundary conditions. As has been shown in [9], the separation between the resonator and the central conductor of the CPW, as well as the distance between the edges of the triangle and the ground plane, must be equal to provide a symmetric boundary for the resonator, favoring a simpler design procedure.

An additional consideration is related to the fact that symmetry must also be used for the boundaries, as demonstrated in [9], where the peak of resonance for a 20 GHz resonator is improved by 5 dB when passing from 25 μm to 50 μm of separation between the triangle edge and the ground plane of the CPW.

**Figure 7:**
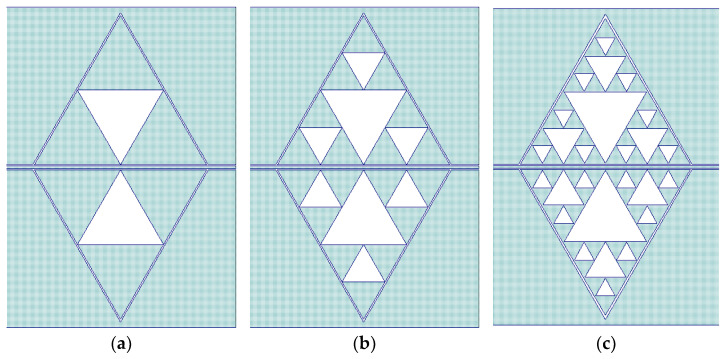

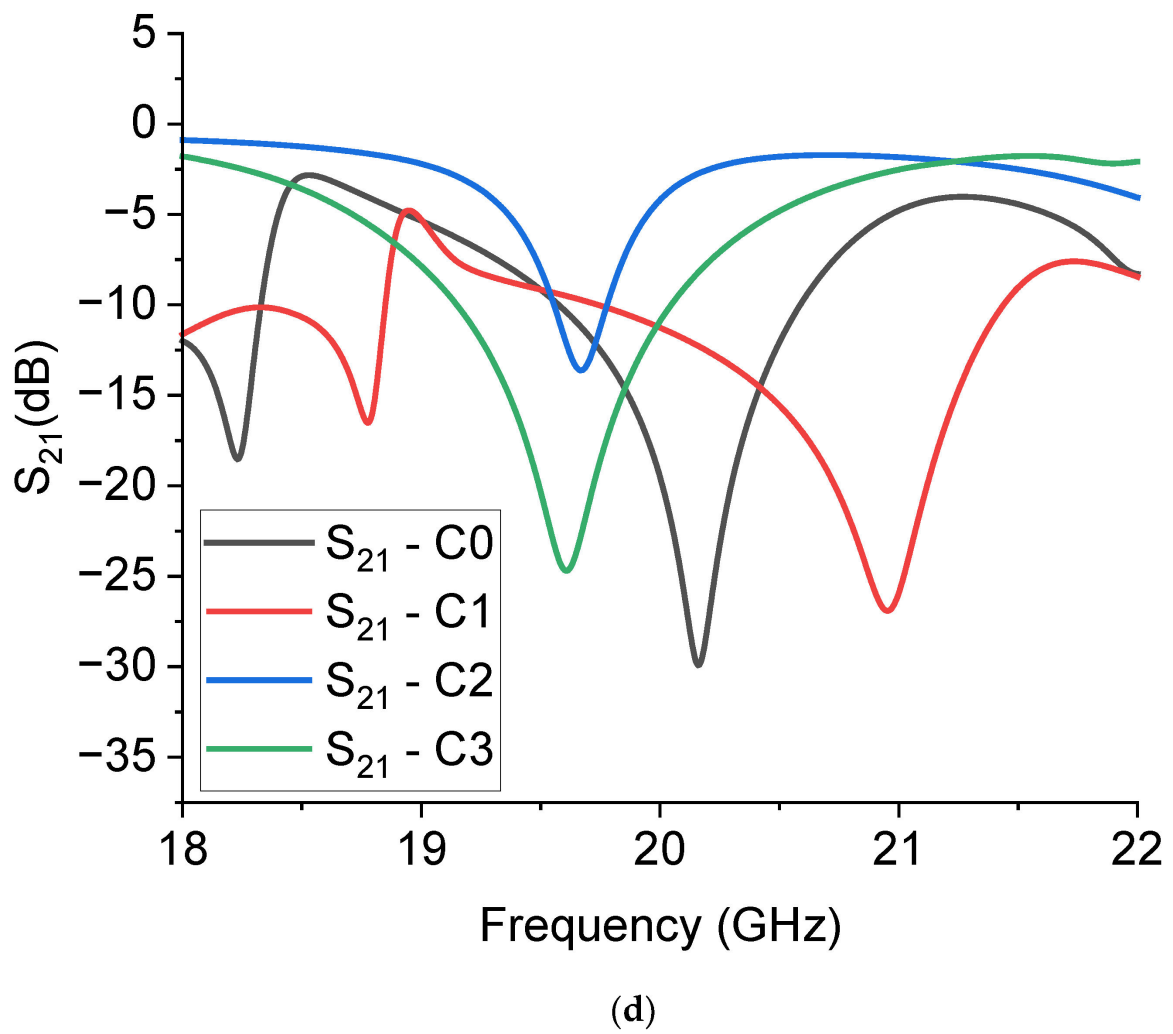
The Sierpinski structures ((**a**–**c**) above) and the simulated transmission parameters ((**d**), S_21_ in dB scale) for the C1, C2, and C3 resonators (below), compared with the response of the reference structure C0.

Following these preliminary considerations, conventional and non-conventional Sierpinski structures have been designed by increasing the internal complexity or combining different levels of complexity. Figure 7 shows the structures and the expected responses for C1, C2, and C3 compared with the reference C0 around 20 GHz.

Analysis of the expected performance of the above resonators allows for important conclusions. The same footprint, reshaping the internal geometry, i.e., changing the level of complexity for the resonator, allows for tuning of the frequency around a chosen value obtained using an electromagnetic simulation. The predicted value for the frequency of resonance *F_resonance_*, from the simulations, is given in Table 3, from which, as well as from Figure 7, it is not clear to obtain a law for *F_resonance_* as a function of the internal complexity.

It is worth noting that, despite the coplanar configuration, and the impossibility of directly using Equation (1), the expected value for *F_resonance_* is now in agreement with the aim of our work to have resonance frequencies close to 20 GHz.

**Figure 8:**
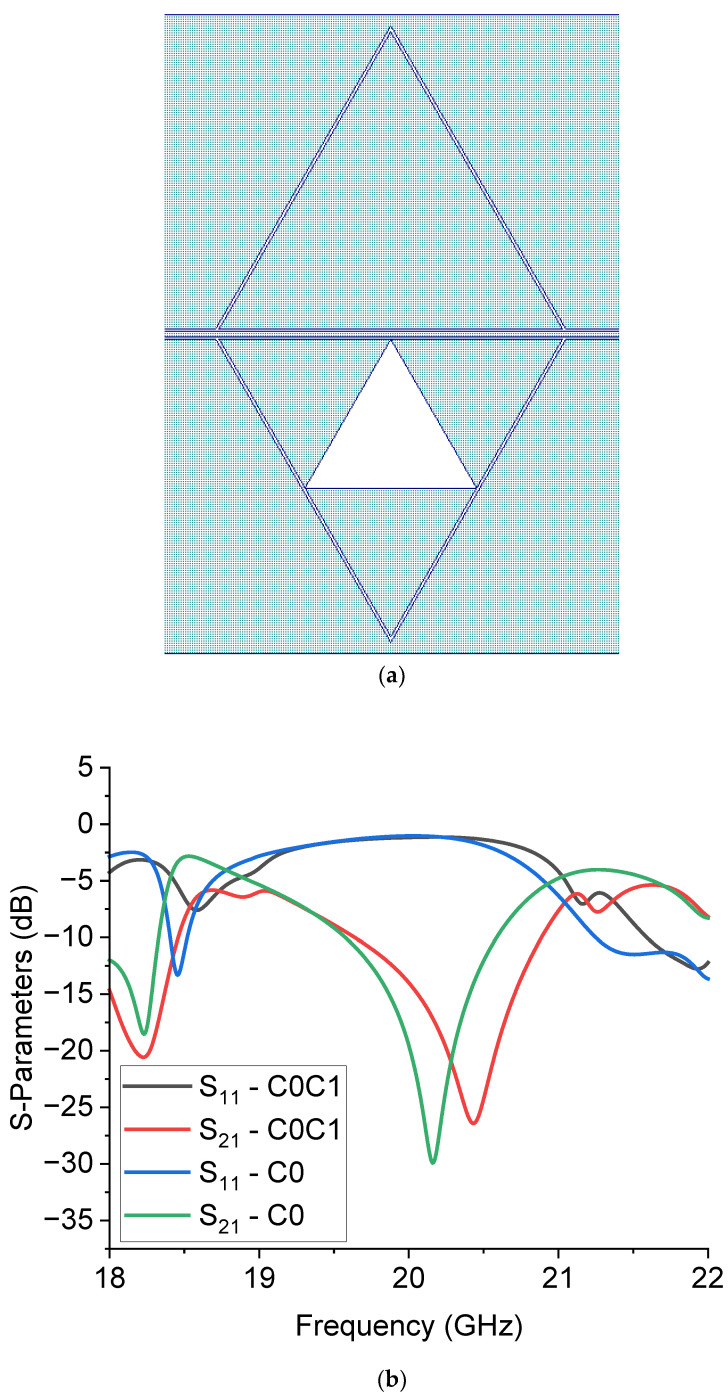
Configuration C0C1 (**a**), made using a C0 triangle interfaced with a C1 one, and its predicted performance (**b**) compared to the pure C0 configuration. Both transmission (S_21_) and reflection (S_11_) parameters are drawn to give evidence for the expected electrical matching of the resonator.

Based on the previous considerations, the correct frequency of resonance can be determined by calibrating the design procedure in conjunction with the experimentally measured value of resonance for the full triangle C0 and the Sierpinski ones. Other resonances in the spectrum will also depend, in terms of position and amplitude, on the feeding system and the coupling mechanism. In principle, a connection between different resonators allows for the selection of adjacent resonance frequencies. A non-conventional way to use the internal complexity is obtained using different triangles symmetrically placed on the two sides of the CPW, as shown in Figure 8, where the structure C0C1 is designed with a C0 triangle on the upper side and a C1 one on the lower side. This arrangement provides an intermediate resonance frequency between C0 and C1. Owing to the small difference in the resonance frequencies for C0 and C1, one of the natural consequences is the bandwidth enlargement of the filter. The same occurs if the complexity increases and the bandwidth is enlarged by increasing the number of internal triangles. Such a finding is clearly expressed in Table 4, where the 3-dB bandwidth (from the negative peak value) is shown together with the frequency of resonance for the two simulated structures; i.e., C0 and C0C1.

It is worth noting that several resonance modes are expected to be excited, not only the main one, as it occurs for every resonator, but it is not easy to predict at what frequency it will occur nor the amplitude or the electrical matching for each of them. For this reason, it could be necessary to redesign the coupling network more efficiently if high-order modes are required.

Then, despite the prediction of the resonance frequency of the main mode, which is not far from the expected value, the question of the effective excitation of the secondary modes and the prediction of the resonance frequency for the Sierpinski triangles with higher internal complexity remains open. Even the main mode is naturally affected by a shift with respect to the predicted one, but this is understandable considering the presence of a coupling network and its contribution to the equivalent circuit of the complete structure.

## 4. Design of the 20 GHz Coupled Hexagonal Resonators

**Figure 9:**
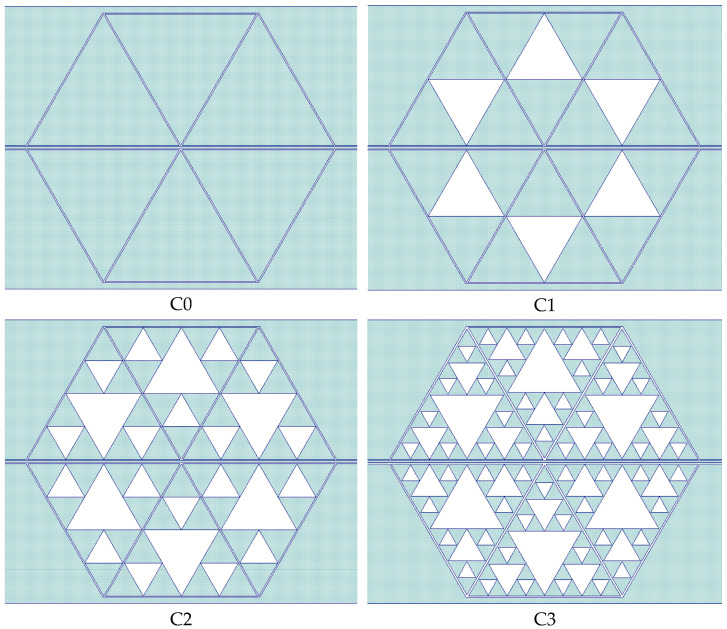
Hexagonal resonators obtained by combining three coupled triangles mirrored with respect to the central conductor of the CPW. All the possible internal complexity levels were designed up to the C3 configuration.

**Figure 10:**
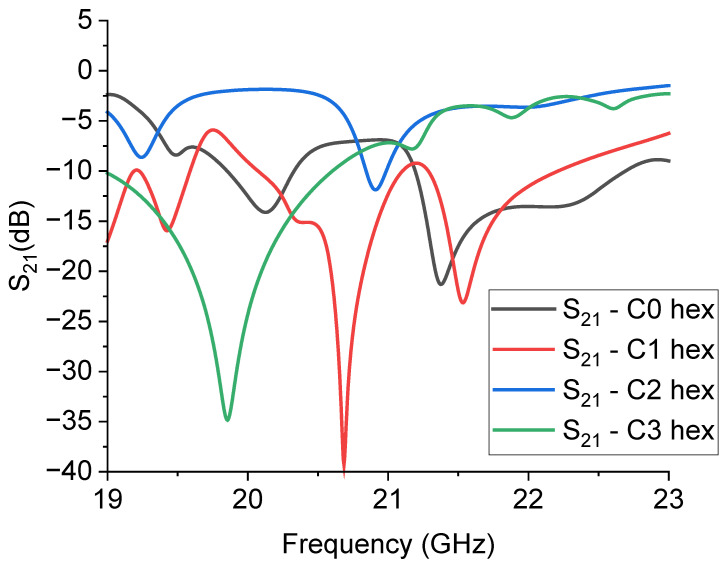
Expected response of the hexagonal resonators, simulated by Microwave Office. The S_21_ parameter is plotted in the dB scale, and the internal complexity is given for each structure with the definition “hex” to indicate that the resonators C0, C1, C2, and C3 were organized in a hexagonal arrangement.

From the good coupling conditions predicted for the single resonators, coupled structures were designed concerning the symmetry criterion for the entire configuration. Three coupled resonators, from C0 to C3, were considered, mirroring the structure with respect to the central conductor of the CPW, obtaining four hexagons with different levels of complexity in this way. The simulated structures and their transmission response are shown in Figure 9 and Figure 10.

Understanding the spectrum of the hexagonal resonators presented in Figure 10 is not a trivial task, because it is evident that the original response of the individual structures has been significantly modified by the coupling among them, and even C0 does not reflect the single resonator response. At this stage, we left the boundaries and the CPW design unaltered in order to make a direct comparison with the previously predicted results. The combination of resonators based on the C1 and C3 geometries is favored for good electrical coupling and, in principle, for the possible choice between two possible bandwidth values. The above results confirm that understanding the coupling mechanisms is very important, analogously to the single resonator design, to obtain a structure potentially useful for improving the electrical matching of the entire structure; i.e., to obtain a deeper notch or specific bandwidths. This result was achieved without further optimizations only in the case of the C1 and C3 structures, with absorption peaks at least 10 dB deeper than those shown in Figure 7 for the individual resonators.

## 5. Design of the 26 GHz Single Resonators

**Figure 11:**
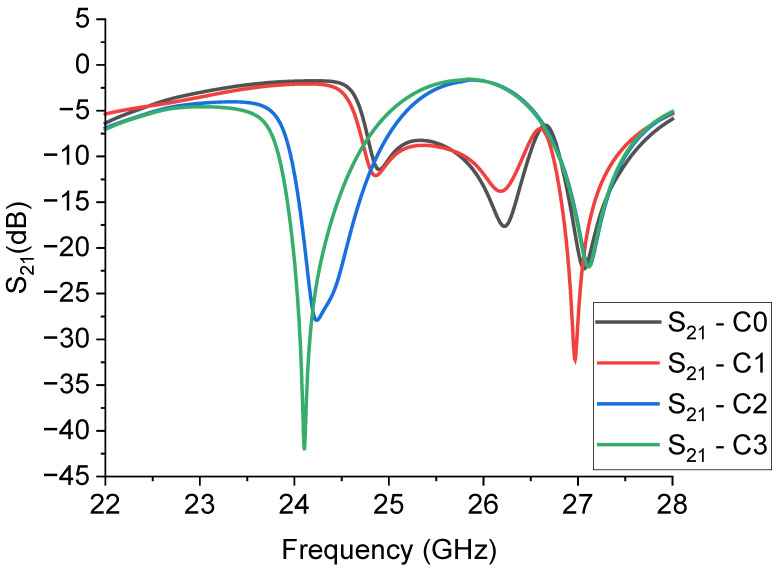
Simulated response of the transmission parameter S21 in dB scale for the small resonators C0, C1, C2, and C3.

**Figure 12:**
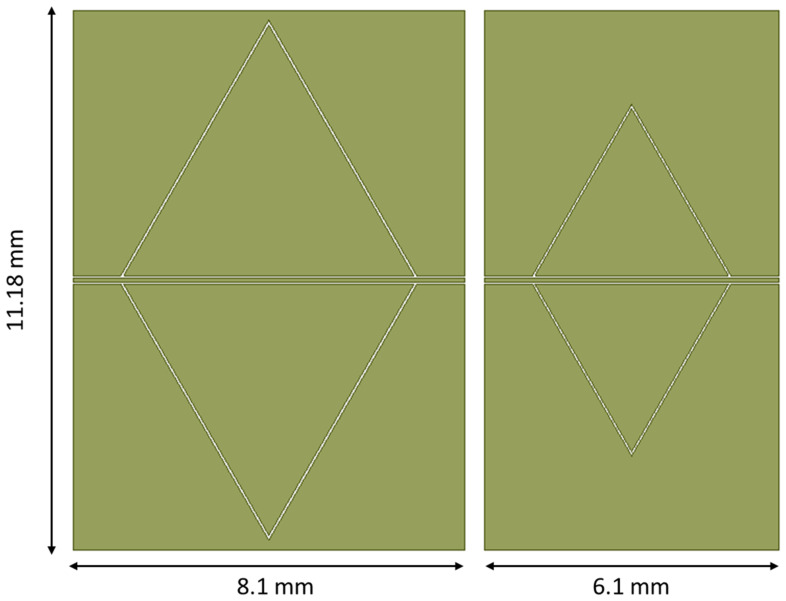
Comparison between the footprint of the big and the small triangular resonators, originally minded for 20 GHz and 26 GHz operations. The side lengths of the triangles are 6 mm and 4 mm, respectively.

The design of the triangles to have a 26 GHz band-stop response presents additional problems in the prediction of the frequency of resonance, as demonstrated in Figure 11, where three mirrored triangles in a CPW configuration were simulated imposing a rescaled edge length, with the same arrangement of the 20 GHz resonators, but smaller size of the triangle. In this case, the longitudinal size of the ground plane remained constant, only changing the length of the central line for the CPW, according to the difference between the edge lengths of the big and the small triangles, as clarified in Figure 12.

It is worth noting that the expected frequency of resonance coming out of the simulations is significantly shifted from the wanted one based on the rescaling procedure and, in this case, the basic configurations C0 and C1 look less promising than C2 and C3. Furthermore, the spectrum of the small resonators appears richer than that of the big ones, and some excited modes are close to each other, with poor selectivity compared to the performance of the big resonators. The modes are excited at approximately 24 GHz and approximately 27 GHz. They are both suitable for K-Band operation, but of course, they need to be properly centered depending on system requirements. On the other hand, exhibiting modes having at least a −20 dB peak is a promising result for dual-band applications. The mixed configuration, involving the simulated structures C2 and C3, will be considered later in the discussion on the experimental results for the 24 GHz filtering capability. The conclusion is that higher frequencies, even if in our case they are not so different between them (20 GHz vs. 26 GHz), might be treated more carefully, including in the design of the optimization of all the geometrical parameters to enhance the mode matching and the filter selectivity. From this point of view, the CPW configuration might be re-designed, also accounting for the boundary ground planes surrounding the resonator.

## 6. Design of the 26 GHz Coupled Hexagonal Resonators

**Figure 13:**
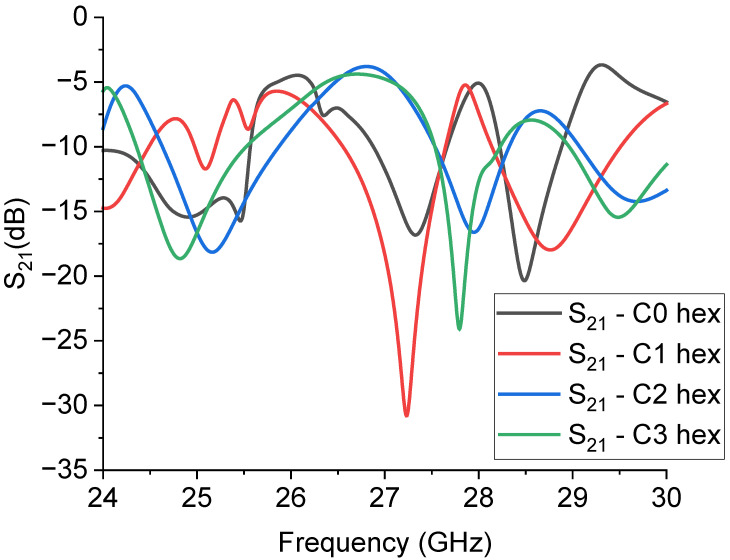
Simulated performance of the hexagonal resonators for operation around 26 GHz. The configuration based on the C1 resonator is the best one in terms of the depth of the notch.

The design of the 26 GHz small hexagonal resonators was performed by simulating the composition of the rescaled triangles in the same way as the 20 GHz resonators. Three resonators were coupled and mirrored with respect to the central conductor of the CPW to obtain a hexagonal arrangement. The simulation is shown in Figure 13, where it is evident that the best performance among them (electrical matching, corresponding to a deeper notch in the resonance) was obtained for the composition of the C1 triangles despite the shift in the expected frequency of operation. This further demonstrates the difficulty in treating the entire structure like the simple composition of single resonators. Looking at Figure 11, where the expected S_21_ of the single resonators is shown, the C1 structure is not necessarily the best one, but it is characterized by a sharp peak around 27 GHz, which is almost the same for the hexagonal resonator but with an enlarged band of operation. An additional optimization for most of the studied structures should concern the electrical matching of the I/O lines.

## 7. Experimental Results and Discussion

### 7.1. Manufacturing of the Devices

The technological solution used for manufacturing the devices was a standard photolithographic process on a high-resistivity silicon wafer. The substrate was 635-μm thick, with a resistivity of ρ≥15,000 Ωcm, and was thermally oxidized at T=1050 °C, to obtain a thickness of silicon oxide of approximately $t_{SiO2}=1\;\mu m$. At high frequencies, this is normally enough to confine most of the electromagnetic field in the wafer’s plane to the dielectric layer’s level. Thermal oxidation helps to provide a dielectric layer in the planar region where the electromagnetic field must be confined to minimize the resistivity losses. The dielectric constant of the obtained thermal oxide was approximately $\varepsilon_{SiO2}=4\pm 10\%$. To mitigate skin depth effects causing losses along the propagation path of the signal, the Au-made planar structures were electroplated with a maximum thickness of 5 μm. The same approach was followed to manufacture all of the studied structures onto the same wafer, including the 20 GHz and 26 GHz resonators. A 200-μm thick frame was left to surround the external figure and the internal triangles, in order to consider the internal complexity of a kind of perturbation with respect to the full triangle and to check the possibility of frequency tunability induced by this choice.

### 7.2. Measurements

The measurements were performed using an on-wafer characterization setup, composed of a Karl-Suss PM5 Probe Station mounting GSG (ground-signal-ground) |Z|-Probes, connected to an HP8510C vector network analyzer system working up to 50 GHz.

**Figure 14:**
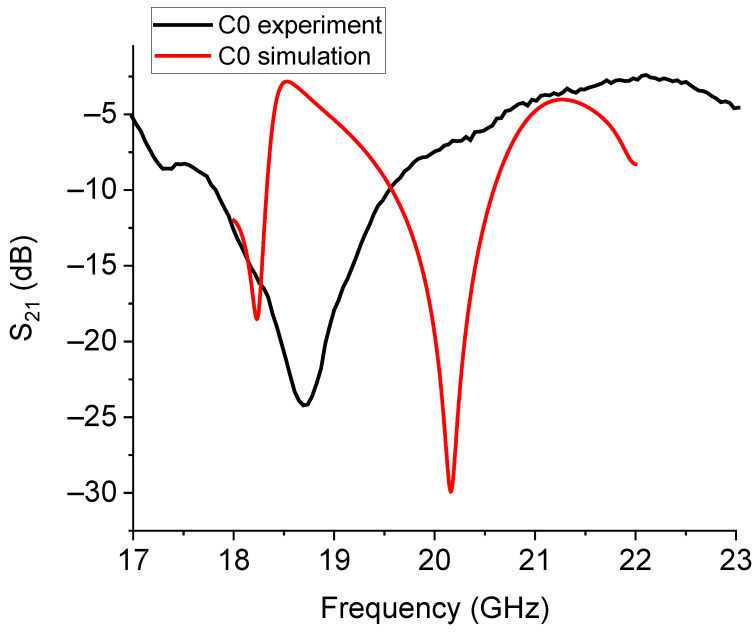
Theoretical and experimental response for the C0 resonator. A significant shift in the expected frequency of resonance and an enlarged bandwidth were obtained.

**Figure 15:**
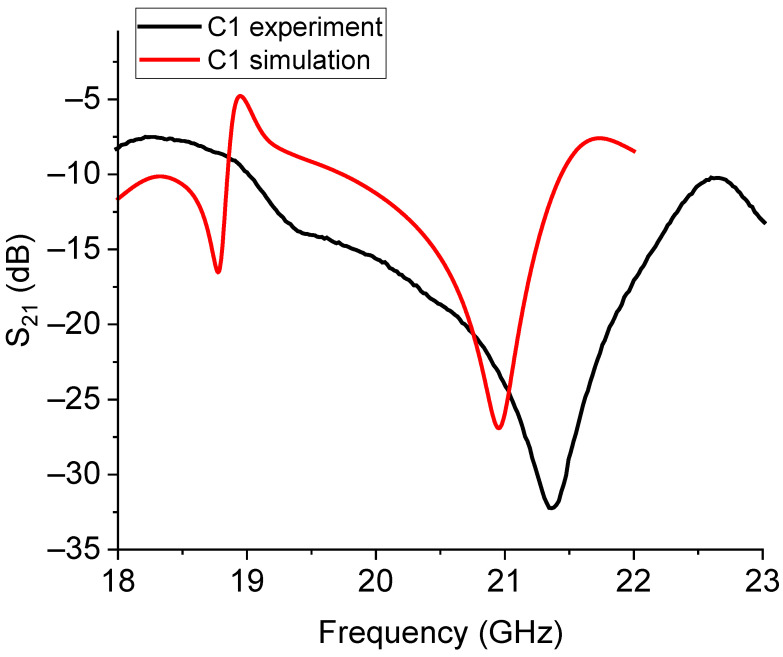
Simulated and experimental response for the C1 Sierpinski resonator.

The single resonators nominally working around 20 GHz and named C0 and C1 were measured, with the results shown in Figure 14 and Figure 15, respectively.

The experimental responses for C0 and C1 were significantly shifted in comparison with the expected resonance frequencies. This agrees with the previous discussion about the difficulty of exactly calibrating the frequency of resonance owing to additional elements to be considered in the equivalent circuit. Further contributions should include the feeding network and the boundaries in order to properly model the resonator response. The measured enlargement of the bandwidth is not necessarily a negative characteristic, but it must be considered in some detail to understand how to control it.

**Figure 16:**
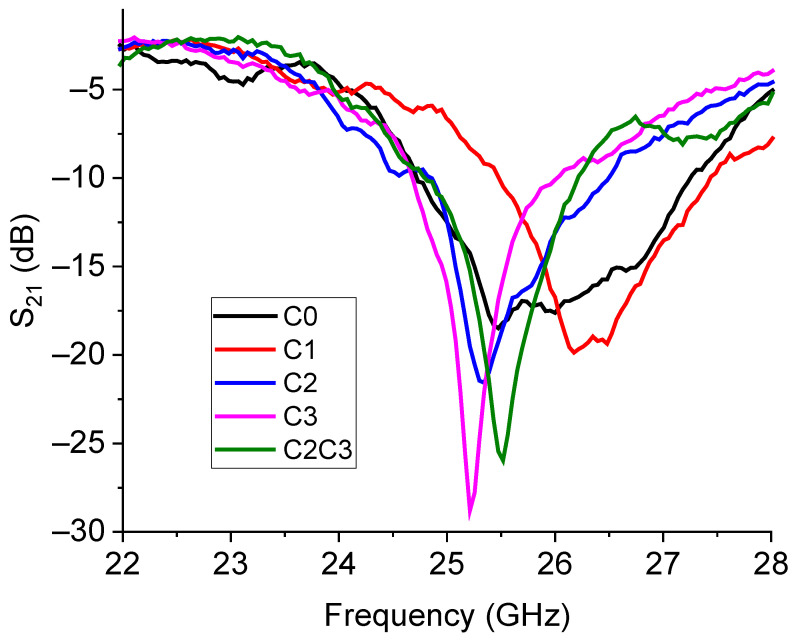
Experimental response for the transmission parameter S_21_ (in dB scale) of the C0, C1, C2, and C3 resonators for the operation at approximately 26 GHz. In addition, the response of the mixed configuration C2C3 was also measured and was shown and compared with the performance of the other resonators.

Resonators designed to work around 26 GHz have also been manufactured and characterized. As discussed in the design section, we expected well-pronounced resonances, especially for the configurations C2 and C3, with deeper notches compared to C0 and C1. To demonstrate the expected better performances at these frequencies of the devices C2 and C3, we first measured the pure configurations from C0 to C3, obtaining the results plotted in Figure 16.

**Figure 17:**
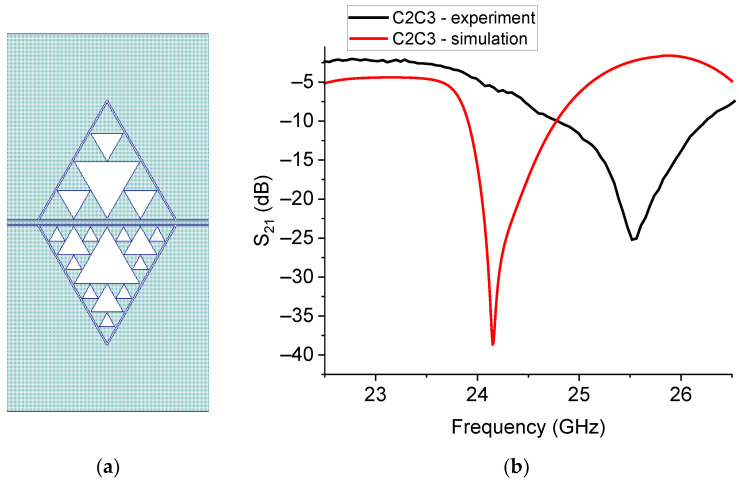
(**a**) C2C3 structure, with the geometry C2 on the top and C3 on the bottom of the resonator, and (**b**) its simulated response for the S_21_ parameter compared with the experimental measurement.

From the plot in Figure 16, it appears that the poor performances expected for C0 and C1 are confirmed, whereas a good electrical coupling is exhibited by the configurations C2 and C3, and by the mixed C2C3 resonator. A more detailed analysis of the C2C3 configuration is shown in Figure 17, where the simulated structure, the predicted response, and the experimental results are shown.

Except for the usual frequency shift, to be calibrated for application purposes, the proposed resonator exhibits quite good performances, with a peak lower than −20 dB, and in this case, low losses along the line.

**Figure 18:**
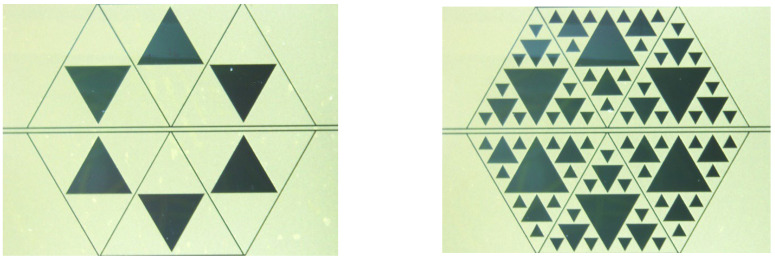
C1 (**left** side) and C3 (**right** side) structures arranged in the hexagonal configuration. The photo is for the 26 GHz resonators, but the geometry is the same as the 20 GHz structure.

Following the simulations presented in the previous sections, 20 GHz and 26 GHz hexagonal structures have been manufactured and tested including the most promising C1 and C3 configurations. The example of the 26 GHz composition is shown in the photo in Figure 18, but the shape is the same as the structures for the 20 GHz operation, which, of course, are more extended than those in Figure 18.

**Figure 19:**
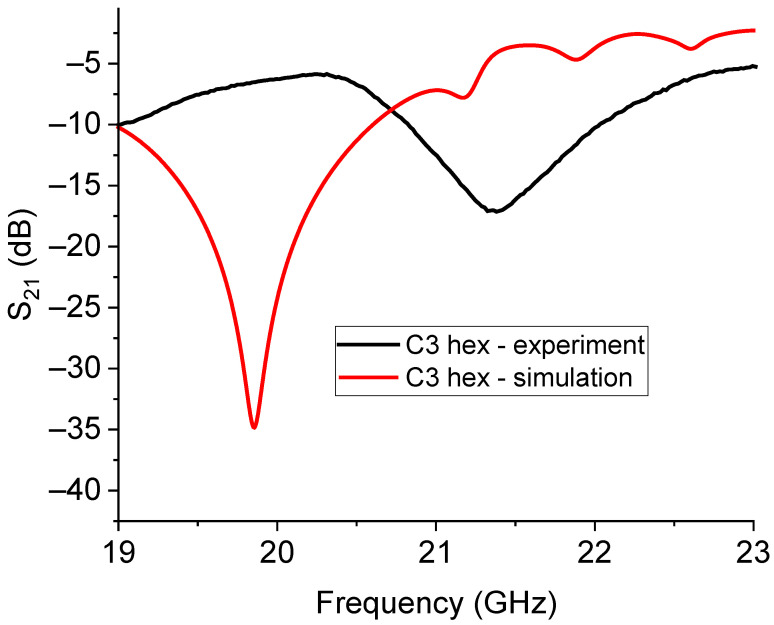
Experimental response of the C3 hexagonal resonator around 20 GHz.

The response for the C3 structure is plotted in Figure 19, comparing the simulation and experimental measurement.

In this case, the structure based on the resonator C3 was not as effective as predicted, because the resonance peak was not only frequency-shifted compared to the simulation, but it was also less pronounced than the expected one. The reason for this disagreement is probably due to some criticalities in the coupling structure. In fact, the number of resonators included in this configuration was high, and the occupied area was extended, with 24 mm of length involved in the coupling of the internal resonator with the external ones considering both sides of the CPW line; this renders it difficult to neglect inhomogeneities in the slot between the resonators, which are not typically considered in the simulations. The electroplating used to increase the thickness of Au for the planar structures to mitigate the skin depth effects was controlled within 10%, and we already evaluated, in [13], the influence of decreasing the gap between the resonator and ground plane to the depth of the peak and, consequently, to the electrical matching. In fact, the electroplating along the edge resulted in a non-perfect vertical alignment of the Au at the end of the process. At the same time, in a 2D simulation, this effect is normally neglected. It should be considered at least with an effective distance between the Au electroplated edges facing each other, using experimental data on the Au final profile. Additional evaluations are needed to optimize this structure, accounting for possible technological limitations, especially when several structures are coupled, and they will be the aim of successive efforts by our group.

**Figure 20:**
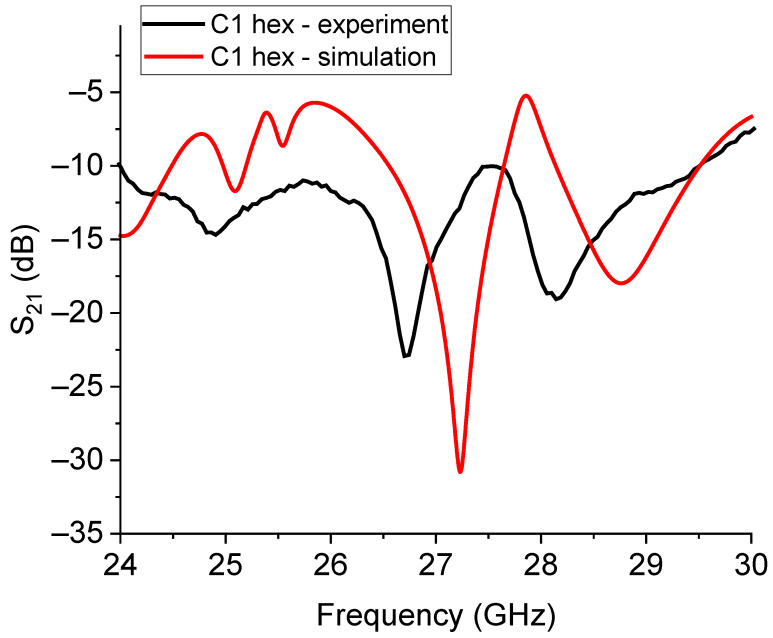
Simulated and experimental responses for the small hexagonal resonator based on the C1 Sierpinski triangles. The label “hex” defines the hexagonal arrangement of the C1 structures. The multi-peak expected response was experimentally measured with a shift in the frequency of resonance of approximately 500 MHz.

**Figure 21:**
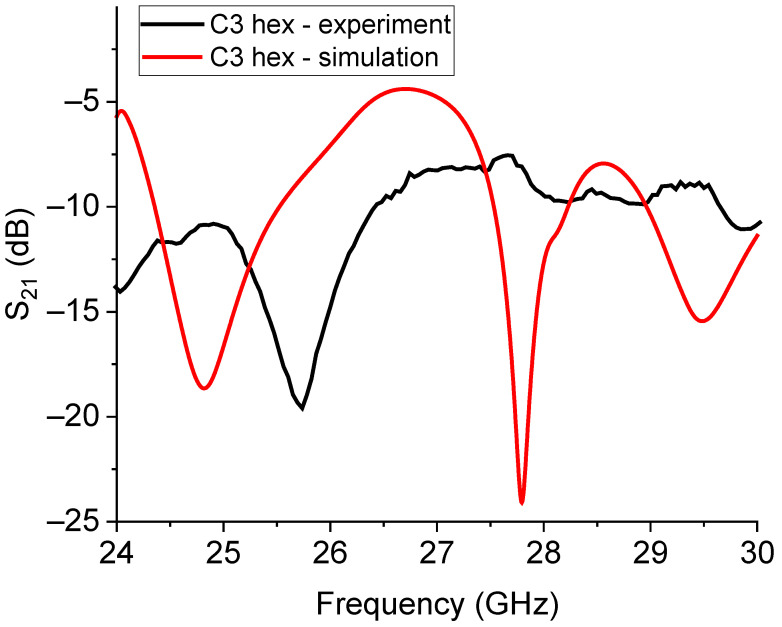
Measured and simulated responses of the small hexagonal resonator based on the C3 Sierpinski configuration for operating frequencies around 26 GHz. In this case, the expected frequency of resonance is around 25 GHz, but the actual frequency is close to 26 GHz. Additional expected modes were not recorded in the experimental measurements.

The expected and experimental responses for the C1- and C3-based hexagonal 26 GHz hexagonal resonators are plotted in Figure 20 and Figure 21. Furthermore, in this case, we can see that the prediction of the resonance frequency is only approximately in agreement with the measured one, and even if the resonators are smaller than those working at 20 GHz, a higher frequency can produce additional complications in the exact modeling of the coupling mechanisms, encompassing the amplitude of the peaks and their frequency position. It is worth noting that the multi-resonance response was correctly predicted for C1, and it just shifted in comparison with the experiment, while for C3, the higher modes around 28 GHz and 30 GHz are suppressed.

From the analysis of the experimental data, it appears that the proposed Sierpinski configurations are promising for the K-band operation, and they generally fulfill the requirements of good electrical matching, as well as having a moderate bandwidth extension when mixed configurations are considered. They are also suitable for coupling between them to form a larger resonator characterized by a transmission response with deeper notches (i.e., multiple poles at the same frequency of the filter response) and presumably better power handling because of the larger metal area. On the other hand, the engineering phase for these devices needs an improved I/O design to decrease the baseline losses. As an additional comment, systematic simulations and measurements are still needed to contribute to the spectrum reconstruction, which is affected by the geometry of the resonator and by the Sierpinski internal complexity, not only for the first mode but also for the secondary ones that are sometimes measured but not predicted by the 2.5D simulation. The modeling of the coupling between adjacent resonators needs further improvement, including, at least using effective quantities, the expected changes in the reciprocal distance introduced by technological processes such as the Au electroplating.

## 8. Conclusions

This paper proposes triangular-shaped band-stop resonators using the Sierpinski geometry for narrow-band microwave tuning around 20 GHz and 26 GHz; i.e., for K-Band applications. The K-Band frequencies are particularly appealing for systems implementing RADAR and satellite communications. As a novelty, fractal Sierpinski geometry was chosen to design, manufacture, and test planar resonators in a coplanar waveguide configuration (CPW).

Sierpinski triangular patch resonators were proposed as building blocks for individual or coupled structures, taking advantage of the possibility of obtaining: (i) different frequencies of resonance maintaining the same footprint but enhancing the internal complexity of the structure, and (ii) arrays of resonators with size reduction, improved electrical response, and better integration compared to square or circular geometries.

Advantages and critical issues in using the CPW excitation for the Sierpinski triangles were widely discussed in our contribution, giving evidence for the difference between the novel CPW structures and those excited by a microstrip line. In particular, the formal approach available in the literature to predict the frequency of resonance for triangles was developed for antennas fed by microstrips or via holes. However, our devices differ from the classical ones due to the CPW embedding, including feeding and boundaries, and the resonance frequency cannot be straightforwardly predicted. At this stage, the prediction of the frequency of resonance can only be obtained by combining electromagnetic simulations with experimental findings. Further electromagnetic simulation efforts linked to experiments will be necessary to build up equivalent circuits and analytical formulations to predict the spectrum of a CPW-fed Sierpinski microwave resonator.

The peculiar results of our research activity concern the necessity to respect symmetrical boundaries for the single resonators and their mutual coupling to obtain acceptable electrical performances for the developed devices. Moreover, coupled structures exhibit improved electrical matching. On the other hand, optimization is still necessary to improve the coupling for all the Sierpinski configurations, considering all the possible internal complexities, as only some of them are immediately suitable for integration.

Bandwidth control can be obtained using mixed configurations designed with mirrored half-triangles characterized by different internal complexity within the same structure.

In the end, promising results have been obtained using partially optimized configurations, but efforts are still needed to fully study the spectral response and coupling conditions.

## Figures and Tables

**Table 1:** Frequencies of resonance, in GHz, for the C0 configuration, comparing the experimental results of [12] with the simulations obtained using CADENCE Microwave Office (MWOffice) release 22.1.

Resonance Frequency for C0 (MWOffice) [GHz]	Resonance Frequency for C0 from Ref. [12] [GHz]	% Error on the C0 Frequency of Resonance
1.290	1.280	0.008
2.255	2.242	0.006
2.615	2.550	0.025
3.440	3.400	0.012
3.850	3.824	0.007

**Table 2:** Frequencies of resonance, in GHz, simulated by Microwave Office for the C0 configuration, and the corresponding resonances obtained for the CPW-fed structure. Only an approximate agreement can be obtained.

Frequency of Resonance for C0 (Microstrip) [GHz]	Frequency of Resonance for C0 (CPW) [GHz]
1.290	0.970
2.255	1.930
2.615	2.915
3.440	?
3.850	3.925
4.660	4.870

**Table 3:** Predicted frequency of resonance, in GHz, for the 20 GHz Sierpinski resonators.

Frequency of Resonance (*F_resonance_*) [GHz]			
C0	C1	C2	C3
20.160	20.950	19.670	19.610

**Table 4:** Comparison among the simulated frequencies of resonances and the 3-dB bandwidth for the configurations C0 and C0C1. Both quantities are in GHz.

	C0	C0C1
*F_resonance_* [GHz]	20.16	20.95
Bandwidth [GHz]	0.096	0.178

## Data Availability

Raw data available upon requirement.
